# A Plain Talk with the Readers of the Journal

**Published:** 1858-07

**Authors:** N. S. Davis


### EDITORIAL.

### A PLAIN TALK WITH THE READERS OF THE JOURNAL.

At the recent annual meeting of the Illinois State Medical Society, the following resolution, in relation to this Journal and its management, was unanimously adopted; and I shall make it the occasion for communicating to the patrons of the Journal a few plain but important facts.

*Resolved*, "That it is the sense of this Society that the CHICAGO MEDICAL JOURNAL is ably conducted, and worthy of the patronage of the profession. That the editors of said Journal be respectfully requested to enlarge their Journal, believing that it would greatly enhance its usefulness; and that each member of this Society should use his utmost efforts to increase its circulation."

The *North-Western Medical and Surgical Journal* came fully under the control of the undersigned at the commencement of its fourth volume, in January, 1855. The purpose was then announced to enlarge the Journal so soon as the receipts from subscribers would enable me to meet the expense.

But instead of finding the receipts in any degree proportioned to the number of subscribers, my first year's experience resulted as follows:

| | |
|---|---|
| Actual cost of publishing, $100 per month, | $1200 00 |
| Actual receipts from subscribers, | 637 00 |
| Cash *out* of my own pocket, | $563 00 |

And yet the list of subscribers on the books as delivered to me, and to whom the Journal was faithfully sent during the year, was near eleven hundred. At the close of that year the subscription list was closely examined, and over two hundred names crossed out as *obsolete.*

The second year the finances of the Journal summed up as follows, viz:

| | |
|---|---|
| Actual cost for the year 1856, | $1200 00 |
| Actual cash receipts from subscriptions, | 877 00 |
| Cash out of my pocket, | $323 00 |

My third year's experience resulted more favorably, and is as follows:

| | |
|---|---|
| Cost of publication for year 1857, | $1200 00 |
| Cash receipts from subscribers, | 1133 00 |
| Loss, | $67 00 |

One-half of the present year has already passed, and with the following result:

| | |
|---|---|
| Cost of publication, first half of 1858, | $600 00 |
| Cash received to July 1st, | 444 00 |
| Loss, | $156 00 |

Thus, during the three years and a half past, the publication of the *Medical Journal* has been a pecuniary loss to me personally of $1109, and that too while the subscription list has been uniformly sufficient (had the subscribers paid) to have met all expenses, and afforded me an income of $600 or $800 per annum, as a compensation for the time and labor bestowed upon it. Such is an exact and plain statement of the pecuniary pleasures and emoluments of publishing a medical journal. When the Journal first came into my hands, its finances were in a state of perfect chaos. Almost every mail brought complaints about erroneous bills, money sent and not credited, etc., and it took nearly all of the first year to get these complaints satisfactorily adjusted. During the last two years, however, not a word of complaint has reached me from any of our subscribers. On the contrary, if compliments and assurances of approbation would pay for paper and printing, I should have had no cause for embarrassment. The subscription list has been slowly but steadily increasing, and we are now sending out regularly every month about 1100 copies. The present number commences the last half of the current year, and yet only 160 of the 1100 subscribers have paid for this volume; and a very large number owe for one, two and three preceding volumes. Hence, there is due me from the subscribers to the *North-Western* and *Chicago Medical Journals* the aggregate sum of $5000.

And yet no *one* of them owes more than $8 or $10.

How *trifling* is the amount for each one, but how vastly important is the aggregate to me? With such an exhibit of financial matters, I might with much propriety decline acceding to the request of the State Society so far as relates to the enlargement of the Journal. For surely none of the members of that Society would ask me to furnish to the profession of the North-West more paper and ink, and pay for them out of my own private income. Notwithstanding all the discouragements, however, I have deliberately resolved upon the adoption of a new system. In compliance, not only with the request of the State Society, but with my own sense of propriety, *sixteen* pages of reading matter have been added to the present number, and the same will be continued in all succeeding numbers. This makes the Journal one-third larger than before, and adds twenty dollars to the cost of each monthly issue. In making this important change, I shall add nothing to the subscription price, leaving it as heretofore, at the low rate of *two* dollars per annum; but let all parties take notice, that, from this time onward, all subscriptions *must be paid in advance.* No new name will be entered on the mail book until the subscription price for, at least, six months, is actually paid. And all who are now in arrears must settle their accounts before the first of October next, or their names will be stricken from the list of subscribers, and their accounts placed in other hands for collection. Therefore, due notice is hereby given, that, after the 1st of October, 1858, every vestige of the *credit system* will be banished from the business affairs of the *Chicago Medical Journal.*

Concerning the editorial management of the Journal, I can only say that heretofore no pains have been spared to make it a genuine aid to the toiling practitioner at the bedside of the sick, and an efficient agent in elevating the general standard of medical education and ethics. The same objects will still be kept predominant in its management, and with the aid of my colleague in the editorial department, Prof. Byford, I trust its future usefulness will be greatly increased.

N. S. DAVIS.

